# The Human Bocavirus Is Associated with Some Lung and Colorectal Cancers and Persists in Solid Tumors

**DOI:** 10.1371/journal.pone.0068020

**Published:** 2013-06-27

**Authors:** Verena Schildgen, Monika Malecki, Ramona-Liza Tillmann, Michael Brockmann, Oliver Schildgen

**Affiliations:** Kliniken der Stadt Köln gGmbH, Klinikum der Privaten Universität Witten-Herdecke mit Sitz in Köln, Institut für Pathologie, Köln, Germany; Columbia University, United States of America

## Abstract

Human bocavirus is the second autonomous human parvovirus with assumed pathogenic potential. Other parvoviruses are known to persist and even integrate into the host genome, eventually contributing to the multi-step development of cancer. Human bocavirus also persists in an unknown percentage of clinically asymptomatic patients in addition to those with primary infection. The aim of the present study was to analyze the role of Human bocavirus in lung and colorectal cancers. Therefore, formalin-fixed, paraffin-embedded, archived tumor samples were screened for Human bocavirus DNA by PCR, Southern blotting, and sequencing. Positive tissues were further subjected to fluorescence in situ hybridization analysis to specifically detect human bocavirus DNA in the infected cells. In total, 11 of the 60 (18.3%) lung and 9 of the 44 (20.5%) colorectal tumors tested positive for human bocavirus DNA by PCR and were confirmed by sequencing and fluorescence in situ hybridization analysis. Thus, human bocavirus DNA is present in the nuclei of infected cells, in either single or multiple copies, and appears to form concatemers. The occurrence of these human bocavirus DNA structures supports the existence of the postulated σ- or rolling-hairpin replication mechanism. Moreover, the fluorescence in situ hybridization patterns inspired the hypothesis that human bocavirus DNA either persists as cccDNA or is integrated into the host genome. This finding suggests that this virus may indirectly contribute to the development of some colorectal and lung cancers, as do other DNA viruses, such as the human hepatitis B virus, or may play an active role in cancer by interacting with the host genome.

## Introduction

Since its discovery in 2005 by Tobias Allander [1], there is increasing evidence that the widespread human bocavirus (HBoV) plays a significant role in respiratory tract infections (subtype 1) and in gastrointestinal infections (subtypes 2-4) [2–6]. Additionally, HBoV is the fourth most common virus detected in respiratory infections [7] and is assumed to be the second parvovirus that is capable of infecting humans with the potential to cause clinical disease. However, its role in gastrointestinal infections is now in doubt based on a novel study from the UK [8].

Previously, our group identified DNA sequences containing “head-to-tail” genome fragments connected by a newly identified linker stretch. After publication, the observation that “head-to-tail” sequences occur for HBoV subtype 1 was confirmed by Kapoor and colleagues, who identified those structures as episomal covalently closed circular (ccc) DNA in patients infected with HBoV subtype 3 [9]. Kapoor and colleagues, as well as groups from China [10,11] and the USA [12], concluded that these cccDNA structures may be viral genomes persisting in these cells. This conclusion supports the hypothesis that HBoV can persist in the infected host and can exist in an asymptomatic but productive state, which in turn could explain the relatively high percentage of asymptomatic patients who shed the virus.

Another example of a DNA virus that persists in a covalently closed circular structure and induces tissue damage is the human hepatitis B virus (HBV). In all chronic HBV infections, cccDNA is present in liver cells, and this cccDNA persistence induces chronic inflammation that does not affect the patient’s condition for a long time but slowly leads to liver-fibrosis, cirrhosis, and finally cancer in a remarkable percentage of chronic HBV infections [13–17].

Given that other parvoviruses are able to integrate into the host genome [18], and the first known pathogenic human parvovirus, parvovirus B19, is associated with several cancers, including lymphomas [19], testicular tumors [20], papillary and anaplastic thyroid carcinomas [21,22], and chronic inflammatory diseases such as Hashimoto’s thyroiditis [23], cardiomyopathy and myocarditis [24], a similar mechanism for HBoV-associated pathogenesis appears possible.

The disease course of HBOV’s closest relatives, i.e., bovine parvovirus (BPV) and minute virus of canine (MVC, CnMV, also known as CPV-1), results from an initial infection of the airway, followed by the infection of the intestine and subsequent shedding via the respiratory/intestinal route [25]. The hypotheses that HBoV can persist in any target organ and that those affected tissues experience long-term damage should thus be tested.

Thus, we addressed the question of whether HBoV, by analogy to the hepatitis B virus, can be detected in tumors. Considering that the pathogenesis of HBoV infections begins in the airways and ends in the gastrointestinal tract, we analyzed non-small cell lung cancer (NSCLC) tumors and colorectal tumors. Both are cancers in which tumor development is poorly understood and in which a viral contribution has not been excluded thus far [26–29].

## Materials and Methods

### Ethics statement

All procedures were performed in accordance with the declaration of Helsinki and according to a vote of the Ethical Committee of the Private University of Witten-Herdecke (vote no. 73/2012). This vote was specifically approved for the current study. Due to the retrospective nature and the double-blinded patient samples used in the study, the Ethical Committee concluded that no written informed consent was required. The study cohort consisted exclusively of adult patients.

### Patient samples

Sixty formalin-fixed, paraffin-embedded (FFPE) lung tumor sections and 44 colorectal tumors were randomly selected from archived samples from our routine clinical laboratory. The mean age of the patients was 69.21 years, with a median of 71 years. The study was performed retrospectively. No further clinical data, including therapy status, smoking behavior, or cancer therapy, were used because that information would have had no influence on the study outcome. As controls, tumor-free tissue from the region of each tumor was analyzed for HBoV whenever available. An additional 10 tumor-free, healthy lung and colorectal tissue samples were analyzed for human bocavirus DNA. As additional controls, samples from 10 human papilloma virus (HPV) positive cervical tumors and 10 HPV negative cervical tumors as well as 10 breast tumors were screened for HBoV DNA. All clinical samples were collected in 2011 and 2012.

### PCR and real-time PCR Analyses

DNA from FFPE tissue samples was extracted using the Maxwell 16 FFPE tissue kit (Promega, Mannheim, Germany) according to the manufacturer’s protocol. PCR and real-time PCR were performed as previously described [30,31].

### Sequencing

Samples that tested positive by real-time PCR for human bocavirus were subjected to conventional PCR using the primers HBoV-LC-Ku-1 and HBoV-LC-Ku-2 [30,31]. PCR-amplified DNA was loaded on a 1.5% agarose gel in TAE buffer and was gel-extracted prior to sequencing. Gel extraction was performed using a Qiagen Gel-Purification kit (Qiagen, Hilden, Germany) strictly following the package insert. For sequencing, purified PCR products were premixed with either the HBoV-LC-Ku-1 primer or the HBoV-LC-Ku-2 primer and sent to Eurofins MWG (Munich, Germany) for capillary Sanger sequencing. The FASTA sequences obtained were subjected to sequence analyses and alignment using Vector NTI software 12.0 (Invitrogen, Karlsruhe, Germany). Sequencing was performed for all tumors that tested positive for HBoV by PCR (n=20).

### FISH Analyses

FISH analyses were performed essentially as described for other commonly tested biomarkers, such as HER2neu and EML4-ALK, as previously published [32], with the modification that HBoV- and GAPDH-specific probes were used, the latter as a control. The probes were designed to hybridize near the two terminal regions of the HBoV genome or to both the 5’-end and the 3’-end of the control gene (GAPDH).

The testing method was similar to the break-apart FISH approach used for the detection of EML4-alk. When two probes bind in close proximity to each other, the red and green signals are close enough to appear as a single yellow signal. This situation could arise if the HBoV genomes occur as covalently closed circular DNA, as described by Kapoor and coworkers [9]. In contrast, distinct signals separated by at least two signal lengths indicate that probes are hybridized at different locations, as in a linear form of the bocavirus genome.

The probes are listed in table 1. FISH was performed as follows: FFPE tumor tissues, surrounding tumor free tissues, and control tissues were cut into 3-µm-thick slices and mounted on slides. As controls, human HepG2 cells were cultured on chamber slides as previously described [33] and transfected with the human bocavirus plasmids p5TRHBoV [34] and pTA-RSV pCR II-TOPO plasmid containing a partial RSV N gene sequence) using Lipofectamine (Invitrogen, Karlsruhe, Germany).

**Table 1:** FISH probes used for the detection of HBoV and GAPDH genes in FFPE slides of colorectal and lung tumors.

**Target**	**Fluorochrome**	**Emission**	**Sequence**	**T_M_**	**Modification**
GAPDH Start	Rhodamine Green	green	TCTACGAGCCTTGCGGGCTCCGGGTCTTTGCAGTCGTATG (40)	>75°C	5'-RGR, 3'-RGR
GAPDH Stop	Rhodamine Red	red	CTGGGGACTGGCTTTCCCATAATTTCCTTTCAAGGTGGGG (40)	74.6°C	5'-RRE, 3'-RRE
Boca Head S	Rhodamine Green	green	TCAGACTGCATCCGGTCTCCGGCGAGTGAACATCTCTGGG (40)	>75°C	5'-RGR, 3'-RGR
Boca Tail S	Rhodamine Red	red	GTTCCTCTCCAATGGACAAGWGGAAAGAAAAGGGTGACTG (40)	72.5°C	5'-RRE, 3'-RRE

All probes were coupled to fluorochromes at their 3’- and 5’-ends to enhance the fluorescence signals.

Slides were processed according to the ZytoLight SPEC ALK/EML4 TriCheck^TM^ probe protocol (ZytoVision, Bremerhaven, Germany) following the manufacturer’s recommendations. Probes were used at concentrations of 2 pM each. Microscopic analyses and documentation were performed on a Zeiss Axioplan microscope using the AxioVision 4.8 software (Zeiss, Jena, Germany). All HBoV positive tumor samples were subjected to FISH analyses (n=20).

## Results

**Figure 1:**
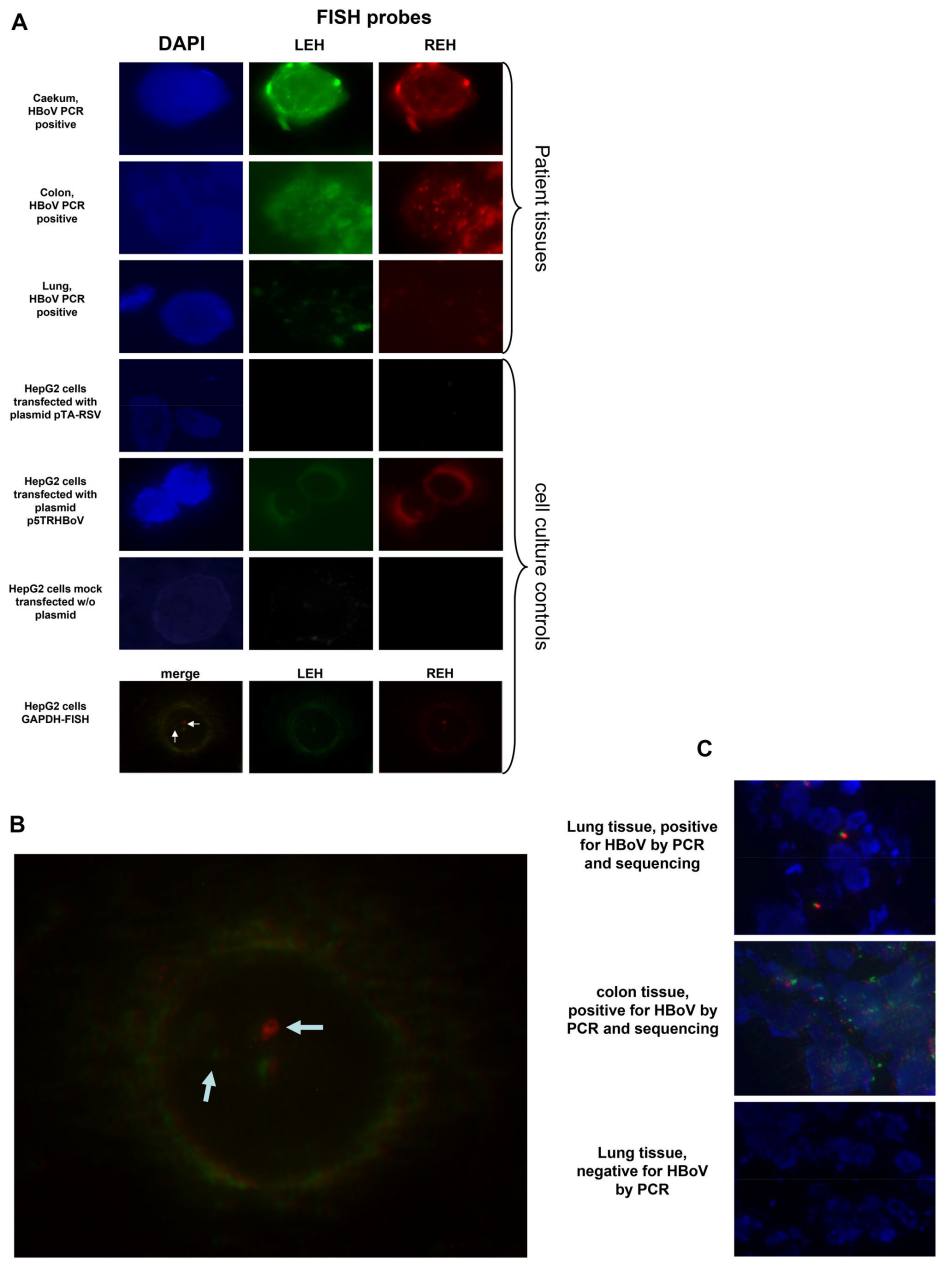
Sequence alignments of HBoV-specific PCR products from tumor tissue samples from lung and colorectal tumors. As reference sequences, the following GenBank accession number were used: HBoV-1=FJ858259(Bonn-1) **a**. aligned PCR product sequences obtained from tumor tissue samples. In total, 15 samples had enough DNA present after PCR amplification for direct sequencing. Sequences were blasted and aligned to the reference strain Bonn-1. It is obvious that all sequences were highly conserved and fully matched the reference sequence. Three sequences had additional non-HBoV sequence upstream of the aligned regions (shown in Figure 1b). **b**. Alignment of the upstream sequence present in three HBoV genomes isolated from tumor tissues. The upstream inserted sequence was observed in three samples that were independently DNA-extracted and analyzed by PCR and sequencing in three independent runs, thus excluding a single artifact or cross contamination event. Surprisingly, the underlined sequences fully matched the human DNA sequence from chromosome 5, reference sequences AC008698.6 (*Homo sapiens* chromosome 5 clone CTB-70H11, bases 76065-76026) and AC025156.2 (*Homo sapiens* 3 BAC RP11-494C5 from Roswell Park Cancer Institute, bases 130245-130284), indicating that HBoV is able to recombine with its host.

In total, 11 of the 60 (18.3%) lung and 9 of the 44 (20.5%) colorectal tumors tested positive for HBoV DNA by conventional end point PCR followed by gel electrophoresis (data not shown) and by qPCR (table 2). The end point and qPCR results were in agreement, i.e., samples that tested positive by end point PCR were also positive by real-time PCR and vice versa, whereas samples that were negative by one method remained negative by the other assay. Furthermore, HBoV DNA was not detected in any of the control group samples (10 breast tumors, 10 HPV-positive cervical tumors, and 10 HPV-negative cervical tumors). Sequencing of the PCR products and subsequent alignments confirmed that HBoV DNA was amplified from the tumor samples (Figure 1a). However, there was no correlation between the HBoV copy number and the tumor stage.

**Table 2:** Patients, tumor types and qPCR results from tumor tissues.

**Patient No.**	**Sex**	**Tumor origin**	**HBoV end point PCR**	**HBoV end point PCR from tumor environment**	**HBoV FISH**	**HBoV copies/µg total DNA**	**Alignment-No. (Figure 1)**
**CR1**	female	appendix	positive	negative	positive	12065	6
**CR2**	Male	caecum	positive	negative	positive	2849	15
**CR3**	female	caecum	positive	negative	positive	885	failed sequencing
**CR4**	Male	colon	positive	negative	positive	12	5
**CR5**	Male	colon	positive	negative	positive	1418	2
**CR6**	female	colon	positive	negative	positive	70	failed sequencing
**CR7**	female	rectum	positive	negative	positive	1710	failed sequencing
**CR8**	Male	rectum	positive	negative	positive	65	failed sequencing
**CR9**	female	rectum	positive	negative	positive	1	failed sequencing
**L1**	female	lung	positive	negative	positive	94804	13
**L2**	female	lung	positive	negative	positive	1563	14
**L3**	Male	lung	positive	negative	positive	3	7
**L4**	Male	lung	positive	negative	positive	11959	4
**L5**	female	lung	positive	negative	positive	3	3
**L6**	Male	lung	positive	negative	positive	336	1
**L7**	female	lung	positive	negative	positive	172857	12
**L8**	female	lung	positive	negative	positive	6190	12
**L9**	female	lung	positive	negative	positive	254000	11
**L10**	Male	lung	positive	negative	positive	16300	8
**L11**	female	lung	positive	negative	positive	11037	9

CR = colorectal; L = Lung

In all HBoV-positive patient tumors (table 2), the healthy tissues surrounding these tumors were also tested for HBoV DNA by PCR (n=30; 2 tumor free FFPE blocks from the area surrounding each HBoV-positive tumor). HBoV DNA was not detected in any of the tumor free tissue samples investigated (two adjacent FFPE blocks per patient, one on each side of the tumor block). Moreover, no HBoV DNA was detected in FFPE tissues from patients without lung or colorectal tumors (10 healthy control lungs and 10 healthy control colorectal tissues). Fifteen isolates passed Sanger sequencing quality control and revealed highly conserved HBoV-1 NP1 gene sequences.

Surprisingly, in three cases, the NP1 DNA sequence was flanked by an upstream sequence that contained a conserved part of human chromosome 5 (1b). As these three samples were subjected to independent DNA extraction, PCR, and sequencing runs, the possibility that this observation resulted from cross contamination among the three samples can be excluded.

As it was previously shown that HBoV can persist in an episomal DNA form that is chemically defined as a covalently closed circular (ccc) DNA, we investigated the form in which HBoV DNA persists in these tumor samples.

**Figure 2:**
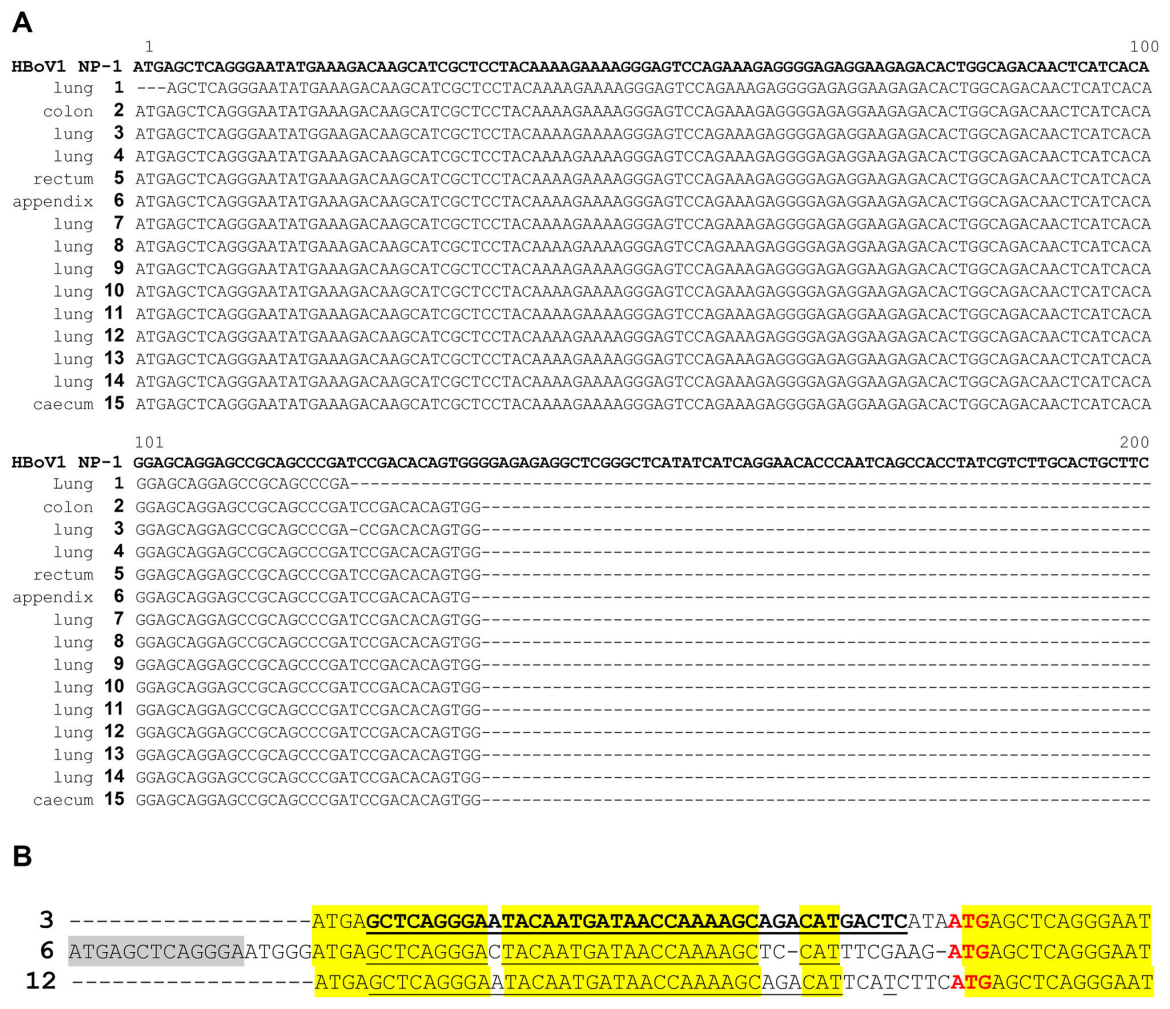
This figure shows selected representative fluorescence microscopy images from HBoV DNA positive tumor samples and control cells. **a**. Rows 1-3 show tissues stained with HBoV-specific probes and DAPI; rows 4-6 show positive and negative controls. LEH and REH correspond to the left end (5’-end) and the right end (3’-end) of the viral genome or the GAPDH gene. Human cells transfected with plasmids with or without human bocavirus genomes as well as mock transfected cells were used as controls. Row 7 shows HepG2 cells stained with probes specific for terminal sequences of the GAPDH gene. **b**. Enlargement of the merged image of HepG2 cell double stained with probes specific for the human GAPDH gene. This figure shows that the distance between the two different probes at the 5’-end and the 3’-end is large enough to result in separate signals (split signal). **c**. Merged images of HBoV DNA positive tissues, including a negative control tissue.

To answer this question, a fluorescence in situ hybridization (FISH) assay was developed. The human HepG2 cell line was transfected with the p5TRHBoV [34] plasmid, which contains a nearly full-length copy of the HBoV genome. As controls, we transfected HepG2 cells with a plasmid containing a partial human respiratory syncytial virus (RSV) sequence and mock-transfected cells. These data are shown in Figure 2a.

FISH probes targeting the regions near the left end hairpin (LEH) and the right end hairpin (REH) were used. One probe was labeled with a green fluorescent dye, and the other was labeled with a red fluorescent dye (table 1).

Transfected cell cultures analyzed by fluorescence microscopy showed that the probes used for the detection of HBoV genomic DNA were highly specific; only those cells that were transfected with p5TRHBoV plasmid and neither the mock transfected cells nor cells transfected with the control plasmid showed signals (Figure 2a, cell culture). These experiments were performed in triplicate.

In all tumor samples that tested positive by PCR, HBoV was also detected by FISH analysis (n=20, table 2). Multiple signals per cell were detected in 3 colorectal tumors and 4 lung tumors, and single signals were detected in the remaining 6 colorectal and 7 lung tumor samples. Figure 2a shows images representing each tumor type observed in the FISH analyses. HBoV DNA was not detected in the negative control samples. HBoV-FISH signals from both the LEH and REH were detected. Surprisingly, their staining pattern was not homogenous: some samples showed a single REH and LEH signal per infected cell, and multiple signals were detected in other samples. Multiple signals per cell were observed in some lung and colorectal tissues (lung: n=4, colorectal: n=3), indicating that multiple copies of HBoV DNA are present in the cells due to replication or multiple genomes persisting as episomes or being integrated into the host DNA. These observations are congruent with the fact that HBoV replicates in a focal manner. Additionally, these signals were not always located in the nucleus; some appeared to be in the cytoplasm, as indicated by merging the different color channels.

Moreover, microscopic FISH analyses of the GAPDH gene shows that the distance between the head and tail signals of the GAPDH gene is large enough to clearly separate both signals (Figure 2a, last line; Figure 2b, enlarged from Figure 2a). Merging the fluorescent images from HBoV positive tumors shows that the terminal HBoV signals are located near each other in some cases, as indicated by green/red signals with a small yellow intermediate region, whereas the red and green signals were clearly separated in others (split signals), indicating that the genome occurs in a non-circular form. The yellow signals support the earlier observation by Kapoor and colleagues that HBoV genomes persist as cccDNA [9]. Representative fluorescent images are shown in Figure 2c.

## Discussion

In our pilot study, which included 60 lung tumors and 44 colorectal tumors, we identified HBoV DNA in 18.3% (n=11) of all lung tumors and 20.5% (n=9) of all colorectal tumors. The data presented suggest that HBoV is associated with some lung and colorectal tumors and confirm the assumption that HBoV persistence is increased in cancer patients [35]. The occurrence of multiple FISH signals within single cells is compatible with the hypothesis that HBoV replicates by the rolling circle or the alternative rolling hairpin mechanism of replication. To investigate this issue, further studies using confocal microscopy, a method that is not yet established in our laboratory, may be helpful.

The HBoV strains identified in tumor samples in the present study all belonged to genotype 1, which is the most frequent genotype in Europe; it is not unusual that no other subtypes were identified, as the other subtypes are generally rare and are associated with acute gastroenteritis, a clinical disorder that was not investigated in our study.

Whether HBoV DNA persists in tumors as episomes or whether it is integrated into the human genome remains to be investigated. Our data show that the HBoV genome can be found in a linear form (split signals) or in a “head-to-tail” form (yellow merged signal or directly adjacent green and red signals). This observation must be interpreted carefully without further analyses. The non-separated signals could represent “head-to-tail” replication intermediates, cccDNA, as observed previously [9,10,36], or multiple genome copies persisting in infected cells. The split signals could be speculatively interpreted as rolling hairpin replication intermediates or as linear genomic DNA. Furthermore, DNA not present in the nucleus could be located in the cytoplasm or packaged into extracellular viral particles.

The finding that HBoV can persist in some infected tissues in the form of cccDNA, similar to other carcinogenic viruses, could be interpreted in several ways. First, HBoV could contribute to the development of some lung and colorectal tumors, acknowledging that it remains unclear whether HBoV has an active or passive role in the development of these tumors. This hypothesis remains a matter of speculation but is supported by the human DNA fragments from chromosome 5 found in three fully independent cases, indicating a recombination event with host DNA (Figure 1b). Though it is unlikely, there is a minimal risk that this result is a PCR artifact. Whether HBoV is able to integrate into human chromosomal DNA or recombine with the human genome remains to be investigated. In a follow up study, this hypothesis could be investigated by using fresh tumor tissue and RFLP analysis followed by Southern blotting, which would lead to a band shift in the case of integration into the host genome. Unfortunately, this was technically impossible in our study because the FFPE tissue was used retrospectively. Furthermore, it is necessary to analyze the expression of viral proteins in the respective tumors and their interaction with cellular proteins, a method that cannot be performed in the absence of widely available antibodies.

Second, some lung and colorectal tumors could provide an optimal environment for HBoV and support HBoV replication. The second assumption in the first instance appears to be more likely because other parvoviruses prefer dividing and proliferating cells [37,38].

The roles of human parvoviruses as causative agents of cancer or contributors to carcinogenesis have been extensively discussed for the first known human parvovirus B19. In 2007, a Chinese group detected parvovirus B19 in 81.1% of colon adenocarcinomas by in situ hybridization and confirmed this result in 78.4% of them by immunohistochemistry detecting viral proteins in the ISH-positive tissues [39]. That study supported an earlier clinical observation in which B19 was associated with lung cancer in a Japanese patient [40]. Moreover, as mentioned in the introduction, parvovirus B19 was suspected to be a causative agent of other cancers and may induce chronic inflammatory processes in an infected tissue [19–24]. Similar mechanisms may play a role in HBoV infected tissues; future studies should thus focus on the inflammatory processes associated with HBoV. Taken together, these observations lead to the hypothesis that human parvoviruses, particularly HBoV and parvovirus B19, may play an active role in human cancers. Therefore, further studies based on prospective studies using fresh tumor tissues or the novel CuFi-8 cell culture system [7] are highly desirable and could elucidate the role of these viruses in human cancers.
